# Peripheral Blood Lymphocyte-to-Monocyte Ratio as a Useful Prognostic Factor in Newly Diagnosed Multiple Myeloma

**DOI:** 10.1155/2018/9434637

**Published:** 2018-11-26

**Authors:** Ying Tian, Yue Zhang, Wan-Qiu Zhu, Xiao-Lei Chen, He-Bing Zhou, Wen-Ming Chen

**Affiliations:** ^1^Department of Hematology, Beijing Chao-Yang Hospital, Capital Medical University, Beijing, China; ^2^Department of Hematology, Beijing Lu-He Hospital, Capital Medical University, Beijing, China

## Abstract

The survival of individuals with tumors may be predicted by the peripheral blood lymphocyte-to-monocyte ratio (LMR) upon diagnosis in recent studies. For patients with multiple myeloma (MM) in the era of novel agents, the prognostic significance of LMR remains unclear. In this study, the prognostic impact of LMR is evaluated by 285 patients with MM who are treated with proteasome inhibitor and/or immunomodulatory drug. LMR is a proven predictor of survival using the receiver operating characteristic curve, with 4.2 as the cutoff point. Patients with LMR ≤ 4.2 at diagnosis had poorer overall survival (OS) and progression-free survival (PFS) than those with LMR > 4.2. In addition, multivariate analysis showed that LMR less than 4.2 is an independent predictor for the OS (hazard ratio [HR]: 1.703; 95% confidence interval [CI]: 1.020–2.842;* P* = 0.042) and PFS (HR: 1.831; 95% CI: 1.098–3.053;* P *= 0.021). According to the test, the LMR at diagnosis, which functions as a simple index reflecting host systemic immunity, can predict clinical outcomes in patients with MM who are treated with new agents.

## 1. Introduction

Multiple myeloma (MM) is the second most common hematological malignancy, which accounts for nearly 10% of all hematological malignant disorders and 0.9% of all cancer deaths each year [1, 2]. For newly diagnosed MM patients, the proteasome inhibitor and/or immunomodulatory drug-based chemotherapy is combined with autologous stem cell transplantation (ASCT) if eligible. Outcome of patients with MM was improved with these new therapies [3]. However, it is worth noting that MM remains incurable, and accurate and practical prognostic indicators for predicting survival will be used for MM patients.

At present, the International Staging System (ISS) [4] is the primary tool to predict the prognosis of MM patients. The Revised-International Staging System (R-ISS) was developed as a newly revised tool in which cytogenetics and lactate dehydrogenase (LDH) are used as the prognostic factors that are independent of the ISS staging system [5]. The R-ISS is proven effective in improving the stratification of patients into more homogeneous risk groups, and it has a better discriminative power than the ISS for MM patients who were treated with novel agents as the primary therapy [6].

The immune system is an important factor in tumor pathogenesis. Immune biomarkers which reflect the baseline host immune status, for example, lymphocyte-to-monocyte ratio (LMR) may be related to the poor prognosis of various tumors [7]. Shi has reported that LMR is associated with the survival of MM patients and is an independent prognostic factor for OS [8]. However, studies on LMR for newly diagnosed MM patients who were treated with novel agents are limited.

This study aimed to investigate the prognostic value of peripheral blood LMR, combined with iFISH in newly diagnosed MM patients.

## 2. Materials and Methods

### 2.1. Patients

285 patients with newly diagnosed MM from the Beijing Chao-Yang Hospital, Capital Medical University, were studied from June 1, 2010 to December 31, 2016. All patients were diagnosed according to the IMWG diagnostic criteria 2014 [9]. The baseline data from the patients were collected: age at diagnosis; gender; complete blood count (CBC); serum albumin, LDH, creatinine, calcium, and *β*2-microglobulin levels; and iFISH analysis including 1q21 gain, del (17p), t (4;14), t (11;14), and t (14;16). The LMR was calculated by peripheral blood white cell count.

The study was censored on December 31, 2017. The study was approved by the ethics committee of Beijing Chao-Yang Hospital.

### 2.2. Statistical Analysis

All patients were treated with at least one novel agent, followed by ASCT if eligible. During the study, the patients were evaluated for CBC, serum albumin, LDH, creatinine, calcium, and *β*2-microglobulin, serum and urine M protein, bone marrow and multiparametric flow cytometry every 3 months. The overall survival (OS) is defined as the time from diagnosis to death from any cause. Progression-free survival (PFS) was calculated from the date of diagnosis to the date of disease progression, death from any cause, or the last contact, whichever occurred first. Kaplan–Meier analysis was conducted to estimate the survival of patients, and log-rank test was used for comparison.* P* values < 0.05 were considered statistically significant. The *χ*^2^ test (nonparametric analysis) was adopted to evaluate the differences between groups. In the multivariate analysis, a Cox proportional hazards model was used. Receiver operating characteristic curves (ROC) and area under the curve (AUC) were used to determine the best cutoff values for survival that is indicated by the LMR. A statistical analysis was performed using the IBM Statistical Package for the Social Sciences software (SPSS Inc, Chicago, IL, USA) or R 3.3.1 software (Institute for Statistics and Mathematics, Vienna, Austria; http://www.r-project.org/).

## 3. Results

### 3.1. Characteristics of the Patients

In this retrospective analysis 285 newly diagnosed MM patients, baseline clinical and laboratory characteristics are listed in Table 1. The median follow-up duration was 48 (range: 2–84) months. The 3-year OS and PFS of the entire cohort were 69.8% and 50.0%, respectively.

**Figure 1:**
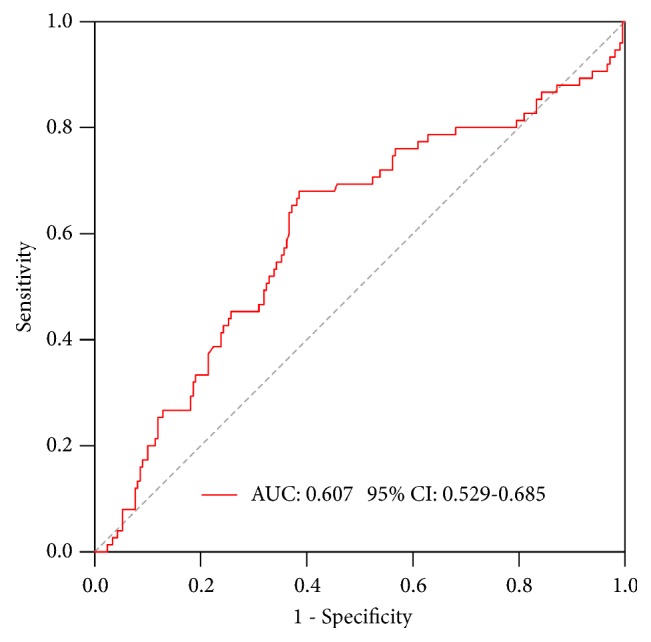
ROC and AUC for the LMR at diagnosis for MM patients. Abbreviations: AUC, area under the curve; LMR, lymphocyte-to-monocyte ratio; MM, multiple myeloma; ROC, receiver operating characteristic.

The median LMR at diagnosis was 6.18 (range: 0.60–118.00). Using the data from the entire cohort, we selected the cutoff points of the LMR for predicting the survival outcomes in the ROC curve analysis. The most discriminative cutoff value of LMR was 4.2, with an AUC value of 0.607 (95% confidence interval [CI]: 0.529–0.685; Figure 1).

Table 1 shows the patients' characteristics according to the LMR at diagnosis. Patients with a LMR ≤4.2 had elevated levels of serum LDH (*P* < 0.001), creatinine (*P *= 0.006), calcium (*P* = 0.003), and *β*2-microglobulin (*P* = 0.035), and they had a higher incidence of relapse (*P* = 0.001) than those with a LMR >4.2. The results also showed that patients with LMR >4.2 had a higher incidence of t (11;14) than those with LMR ≤4.2 (24.8% vs. 13.6%,* P* = 0.018).

### 3.2. Prognostic Impact of the Lymphocyte/Monocyte Ratio at Diagnosis

**Figure 2:**
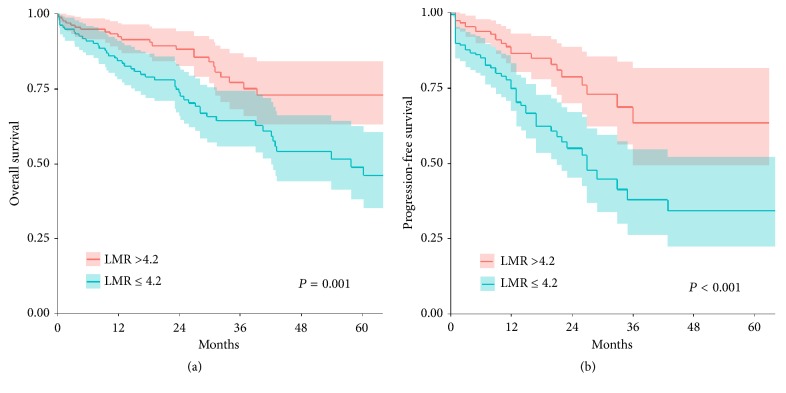
(a) Overall survival for the LMR at diagnosis for MM patients. (b) Progression-free survival for LMR at diagnosis for MM patients. Abbreviations: LMR, lymphocyte-to-monocyte ratio; MM, multiple myeloma.

The OS and PFS of patients with LMR ≤4.2 were significantly lower than those of patients with LMR >4.2 at diagnosis (3-year OS: 64.2% vs. 77.3%,* P *= 0.001; 3-year PFS: 37.9% vs. 68.1%,* P* < 0.001; Figure 2).

Tables 2 and 3 showed the results of the univariate and multivariate analysis of the factors influencing the OS and PFS, respectively. The multivariate analysis revealed that LMR ≤4.2 was an independent prognostic factor for predicting OS (HR: 1.703; 95% CI: 1.020–2.842;* P* = 0.042; Table 2) and PFS (HR: 1.831; 95% CI: 1.098–3.053;* P* = 0.021; Table 3). Moreover, serum *β*2-microglobulin ≥ 5.5 mmol/L was also an independent prognostic factor for OS (HR: 1.810; 95% CI: 1.049–3.125;* P* = 0.033; Table 2) and PFS (HR: 1.758; 95% CI: 1.014–3.047;* P* = 0.044; Table 3).

### 3.3. Prognostic Impact of the Lymphocyte/Monocyte Ratio on Different ISS and R-ISS

**Figure 3:**
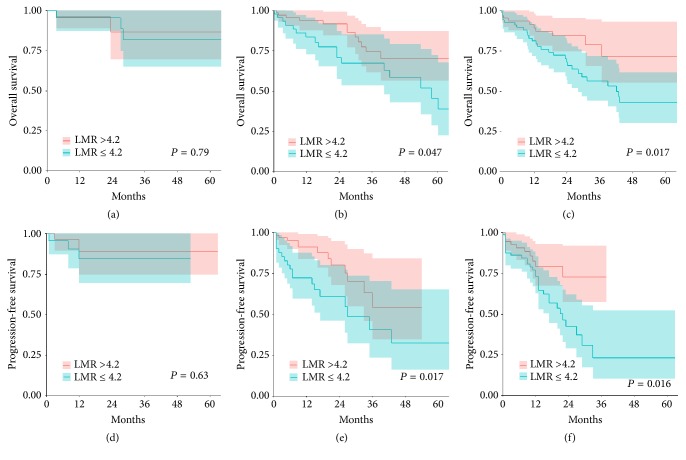
(a) Overall survival in ISS stage I stratified by the LMR. (b) Overall survival in ISS stage II stratified by the LMR. (c) Overall survival in ISS stage III stratified by the LMR. (d) Progression-free survival in ISS stage I stratified by the LMR. (e) Progression-free survival in ISS stage II stratified by the LMR. (f) Progression-free survival in ISS stage III stratified by the LMR.

**Figure 4:**
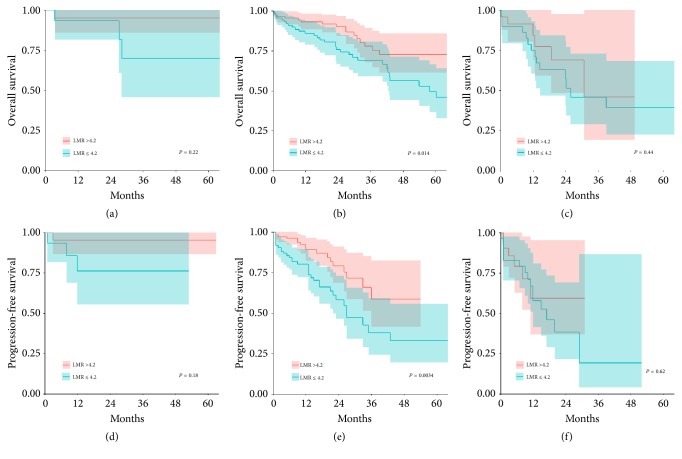
(a) Overall survival in R-ISS stage I stratified by the LMR. (b) Overall survival in R-ISS stage II stratified by the LMR. (c) Overall survival in R-ISS stage III stratified by the LMR. (d) Progression-free survival in R-ISS stage I stratified by the LMR. (e) Progression-free survival in R-ISS stage II stratified by the LMR. (f) Progression-free survival in R-ISS stage III stratified by the LMR.

Further analysis showed that LMR ≤4.2 had a negative prognostic impact on both PFS and OS in ISS stages II and III, while it could not in ISS stage I (see Figure 3). Moreover, LMR ≤4.2 had a negative prognostic impact on both PFS and OS in R-ISS stage II, while it could not in R-ISS stages I and III (see Figure 4).

## 4. Discussion

As a marker of host antitumor immunity, absolute lymphocyte count (ALC) has been widely studied in hematologic and solid malignancies [10]. In MM, ALC recovery after ASCT has significant prognostic value [11, 12]. Moreover, another study has shown that ALC at diagnosis was associated with survival in patients with newly diagnosed MM [13].

The BM micro-environment plays a critical role in the development of MM from its precursor condition, monoclonal gammopathy of undetermined significance (MGUS), in part by allowing immune tumor evasion [14]. In addition, it can support the growth and survival of myeloma cells and influence their migration and drug resistance [15, 16]. Inflammatory cells are essential in tumor progression [17, 18]. Tumor-associated macrophages (TAMs), which constitute a significant proportion of tumor-related inflammatory cells, contribute to the growth, angiogenesis, and metastasis of a variety of tumors [19].

TAMs play an important prognostic role in patients with classical Hodgkin lymphoma (HL) [20–22], follicular lymphoma (FL) [23], and MM [24–26]. Derived from the circulating monocytes, TAMs are recruited to the tumor site by tumor-derived chemotactic factors [27]. Tumor-derived chemotactic factors can influence the number of circulating monocytes and the TAMs, the level of TAM recruitment can be reflected by the peripheral blood absolute monocyte count (AMC), and it may be considered as an important surrogate marker for TAMs. AMC is a poor prognostic factor in diffusing large B-cell lymphoma (DLBCL) [28], FL [29], extranodal natural killer/T-cell lymphoma (ENKL) [30], HL [31], and MM [24].

Based on these results, the ALC-to-AMC ratio may be considered as a symbol of the relative strength of the host immune system to tumor-induced immune dysfunction. A lower LMR has a negative prognostic impact on several solid tumors and hematologic malignancies [7, 32, 33]. A recent study has reported that LMR < 3.6 can be considered a bad prognostic factor for PFS in patients with MM [34]. Therefore, LMR has been associated with the prognosis of patients with several tumors.

In this paper, the prognostic impact of the LMR in newly diagnosed MM patients was treated by proteasome inhibitor and/or immunomodulatory drug-based chemotherapy was retrospectively evaluated. The result showed that LMR was an independent predictor for OS and PFS, and patients with LMR ≤4.2 had significantly elevated serum LDH, creatinine, calcium, and *β*2-microglobulin levels and a higher incidence of relapse. In addition, patients with LMR >4.2 had a higher incidence of t(11;14). However, Dosani et al. have found that LMR independently predicted del(17p) and t(4;14), rather than t(11;14) [35]. This may be due to differences in the study population and may also be related to differences in the selected cutoff values. Thus, prospective studies with a larger sample size must be conducted to validate the results.

Considering the significance of LMR in clinical outcomes, certain tests have also been conducted to validate whether LMR could improve the prognostic impact of the ISS and R-ISS. Interestingly, based on the test results, the addition of LMR to the ISS further defined prognosis, particularly in stages II and III, whereas those to the R-ISS further defined prognosis in stage II. The combination of ISS or R-ISS and LMR improved the predictive value in patients treated with novel agents beforehand.

## 5. Conclusions

The LMR at diagnosis, which is a simple index reflecting host systemic immunity, can predict clinical outcomes in patients with MM who were treated with novel agents. In addition, LMR is an objective and cost-effective test result, and it can be easily obtained from CBC results. Owing to its significant value in clinical treatment, further studies on LMR must be conducted to better understand the roles of peripheral lymphocytes and monocytes in individuals with MM.

## Figures and Tables

**Table 1:** Characteristics at baseline of 285 newly diagnosed MM patients.

Characteristic	All cases (n=285)	LMR ⩽ 4.2 (n=132)	LMR > 4.2 (n=153)	*P*
Age at diagnosis, years				0.478
< 65	197	94	103	
⩾ 65	88	38	50	
Gender				0.200
Male	159	79	80	
Female	126	53	73	
Hemoglobin				0.878
⩾100g/L	126	59	67	
< 100g/L	159	73	86	
Serum albumin				0.073
⩾35g/L	101	54	47	
< 35g/L	184	78	106	
Serum LDH				0.001
< 250U/L	236	96	140	
⩾250U/L	49	36	13	
Serum creatinine				0.006
< 177umol/L	235	100	135	
⩾177umol/L	50	32	18	
Serum calcium				0.003
< 2.75mmol/L	266	117	149	
⩾2.75mol/L	19	15	4	
Serum *β*2-microglobulin				0.035
< 5.5mg/L	172	71	101	
≥5.5mg/L	113	61	52	
1q21 gain				0.851
Negative	155	71	84	
Positive	130	61	69	
del (17p)				0.790
Negative	252	116	136	
Positive	33	16	17	
t (4;14)				0.080
Negative	246	119	127	
Positive	39	13	26	
t (11;14)				0.018
Negative	229	114	115	
Positive	56	18	38	
t (14;16)				0.953
Negative	274	127	147	
Positive	11	5	6	
Relapse				0.001
No	176	70	106	
Yes	83	51	32	
ASCT				
No	196	92	104	0.798
Yes	89	40	49	

Abbreviations: ASCT, autologous stem cell transplantation; LDH, lactate dehydrogenase; LMR, lymphocyte-to-monocyte ratio; MM, multiple myeloma.

**Table 2:** Univariate and multivariate analyses for OS.

Prognostic factors	Univariate analysis		Multivariate analysis	
	HR (95% CI)	*P*	HR (95% CI)	*P*
Age ≥ 65 years	1.045 (0.630-1.733)	0.866		
Hemoglobin < 100g/L	1.305 (0.820-2.078)	0.262		
Serum albumin < 35g/L	1.381 (0.840-2.272)	0.203		
Serum LDH ≥ 250U/L	2.748 (1.661-4.548)	< 0.001	1.775 (0.984-3.199)	0.056
Serum creatinine ≥ 177umol/L	1.953 (1.178-3.238)	0.009	1.138 (0.591-2.192)	0.699
Serum calcium ≥ 2.75mol/L	2.761 (1.371-5.559)	0.004	1.427 (0.657-3.102)	0.369
Serum *β*2-microglobulin ≥ 5.5mmol/L	2.410 (1.523-3.815)	< 0.001	1.810 (1.049-3.125)	0.033
1q21 gain	1.714 (1.086-2.704)	0.021	1.527 (0.957-2.436)	0.076
del (17p)	1.526 (0.803-2.902)	0.197		
t (4;14)	1.009 (0.501-2.033)	0.980		
t (11;14)	1.431 (0.830-2.465)	0.197		
t (14;16)	1.910 (0.695-5.246)	0.210		
LMR ≤ 4.2	2.207 (1.356-3.591)	0.001	1.703 (1.020-2.842)	0.042

Abbreviations: CI, confidence interval; HR, hazard ratio; LDH, lactate dehydrogenase; LMR, lymphocyte-to-monocyte ratio; OS, overall survival

**Table 3:** Univariate and multivariate analyses for PFS.

Prognostic factors	Univariate analysis		Multivariate analysis	
	HR (95% CI)	*P *	HR (95% CI)	*P *
Age ≥ 65 years	1.113 (0.671-1.844)	0.679		
Hemoglobin < 100g/L	1.431 (0.900-2.274)	0.130		
Serum albumin < 35g/L	1.437 (0.874-2.364)	0.153		
Serum LDH ≥ 250U/L	2.820 (1.696-4.687)	< 0.001	1.673 (0.941-2.973)	0.080
Serum creatinine ≥ 177umol/L	2.113 (1.275-3.503)	0.004	1.005 (0.530-1.904)	0.989
Serum calcium ≥ 2.75mol/L	2.996 (1.489-6.032)	0.002	1.499 (0.685-3.278)	0.311
Serum *β*2-microglobulin ≥ 5.5mmol/L	2.541 (1.604-4.024)	< 0.001	1.758 (1.014-3.047)	0.044
1q21 gain	1.772 (1.121-2.800)	0.014	1.586 (0.994-2.529)	0.053
del (17p)	1.439 (0.758-2.731)	0.266		
t (4;14)	1.079 (0.535-2.176)	0.831		
t (11;14)	1.550 (0.901-2.668)	0.114		
t (14;16)	1.638 (0.579-4.496)	0.338		
LMR ≤ 4.2	2.387 (1.469-3.880)	< 0.001	1.831 (1.098-3.053)	0.021

Abbreviations: CI, confidence interval; HR, hazard ratio; LDH, lactate dehydrogenase; LMR, lymphocyte-to-monocyte ratio; PFS, progression-free survival

## Data Availability

The data used to support the findings of this study are available from the corresponding author upon request.
